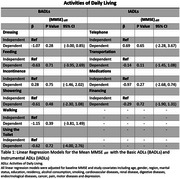# Examining the Hierarchy of Functional Loss in Relation to Cognitive Decline: A 3‐Year Longitudinal Study of Older Adults

**DOI:** 10.1002/alz70858_102125

**Published:** 2025-12-25

**Authors:** Etwal Bou Raad, Celine Verstuyft, Monique Chaaya, Lilian Ghandour Abiad, Emmanuelle Corruble, Laurent Becequemont, Khalil El Asmar

**Affiliations:** ^1^ American University of Beirut, Faculty of Health Sciences, Hamra, Beirut, Lebanon; ^2^ Paris Saclay University, Faculty of Medicine, Le Kermlin Bicetre, Paris, France; ^3^ Paris Sud University, Faculty of Medicine, Paris, Paris, France; ^4^ Centre de recherche Clinique Paris‐Saclay Univeristy, Le Kremlin Bicetre, Paris, France

## Abstract

**Background:**

Many research attempted to explore the hierarchical structure in functional skill decline concerning cognitive deterioration in older adults, however results have been inconsistent. The main objective of this study is to compare cognitive status of participants who have and have not lost independence in particular function skill after three years of follow up. In addition, this study aims to determine whether a hierarchical scale of functional loss is associated with declining cognitive status.

**Methods:**

A cohort of 983 community‐dwelling persons age 65 years and older from S.AGES (Sujets Ages, elderly subjects) were analyzed. At baseline and every 6 months for 3 years cognitive function was further evaluated through the Min Mental State Examination (MMSE) and functional status was evaluated by the activity of daily living (ADLs) including basic (BADLs) and Instrumental ADLs (IADLs). Multivariable linear regression models were used to compare the mean change of MMSE between those who became dependent on a particular functional skill versus those who didn't adjusting for age, gender, alcohol, smoking, baseline MMSE score, motor disorders, CNS diseases and depression.

**Results:**

A total of 680 subjects remained independent in at least one functional skill after 3 years. No hierarchical pattern emerged: participants who lost independence in IADLs did not show a systematically different trajectory of cognitive decline than those who lost independence in BADLs. Likewise, multivariable regression models revealed no significant difference in MMSE decline between those who lost independence and those who did not, across all functional skills.

**Conclusion:**

Further investigations are warranted to examine the natural hierarchy of functional loss associated with cognitive decline as it may be affected by several factors. This information is important for caregivers, clinicians and policymakers as it can anticipate the pattern of functional decline with the subsequent care needs of older adults with declining cognition.